# Antibiofilm Efficacy of the *Pseudomonas aeruginosa* *Pbunavirus* vB_PaeM-SMS29 Loaded onto Dissolving Polyvinyl Alcohol Microneedles

**DOI:** 10.3390/v14050964

**Published:** 2022-05-05

**Authors:** Sanna Sillankorva, Liliana Pires, Lorenzo M. Pastrana, Manuel Bañobre-López

**Affiliations:** INL—International Iberian Nanotechnology Laboratory, Avenida Mestre José Veiga, 4715-330 Braga, Portugal; liliana.pires@inl.int (L.P.); lorenzo.pastrana@inl.int (L.M.P.); manuel.banobre@inl.int (M.B.-L.)

**Keywords:** bacteriophages, *Pseudomonas aeruginosa*, microneedles, polyvinyl alcohol, biofilms

## Abstract

Resistant bacteria prevail in most chronic skin wounds and other biofilm-related topical skin infections. Bacteriophages (phages) have proven their antimicrobial effectiveness for treating different antibiotic-resistant and multidrug-resistant bacterial infections, but not all phages are effective against biofilms. Phages possessing depolymerases can reach different biofilm layers; however, those that do not have depolymerase activity struggle to penetrate and navigate in the intricate 3D biofilm structure and mainly infect bacteria lodged in the outer biofilm layers. To address this, *Pseudomonas aeruginosa* phage vB_PaeM-SMS29, a phage with poor antibiofilm properties, was incorporated into polyvinyl alcohol (PVA, Mowiol 4:88) supplemented with 0.1% (*v*/*v*) of glycerol, and cast onto two different microneedle arrays varying in geometry. The dissolving microneedles were thoroughly characterized by microscopy, force-displacement, swelling, phage release and stability. Furthermore, 48 h-old biofilms were formed using the colony biofilm procedure (absence of broth), and the antibiofilm efficacy of the phage-loaded microneedles was evaluated by viable cell counts and microscopy and compared to free phages. The phages in microneedles were fairly stable for six months when stored at 4 °C, with minor decreases in phage titers observed. The geometry of the microneedles influenced the penetration and force-displacement characteristics but not the antimicrobial efficacy against biofilms. The two PVA microneedles loaded with phages reduced *P. aeruginosa* PAO1 biofilms by 2.44 to 2.76 log_10_ CFU·cm^−2^ at 24 h. These values are significantly higher than the result obtained after the treatment with the free phage (1.09 log_10_ CFU·cm^−2^). Overall, this study shows that the distribution of phages caused by the mechanical disruption of biofilms using dissolving microneedles can be an effective delivery method against topical biofilm-related skin infections.

## 1. Introduction

Bacteria tend to attach to surfaces and form 3D structures, known as biofilms. In biofilms the bacteria secrete a protective extracellular polysaccharide matrix, which glues the cells together, making them inherently resistant to most antibiotics’ penetration and escaping the action of the host immune system [1]. In chronic wounds, numerous antimicrobial-resistant bacteria heavily colonize these wounds, with *Pseudomonas aeruginosa* and *Staphylococcus aureus* being the most commonly isolated [2]. *P. aeruginosa*’s persistence in chronic wounds is associated with more severe outcomes, with wounds generally larger in size and taking longer to heal than wounds where this pathogen is absent [3]. The management of chronic wounds is a pressing global health issue not only due to the rising levels of microbial resistance to conventional treatments and microorganisms in biofilm communities that prolong the infection and lead to chronic infections [4,5] but also due to the lack of viable delivery methods of antimicrobials (reviewed in [6]). 

The topical delivery of antimicrobials to wounds decreases systemic toxicity and side effects generally localized to the application site. However, conventional topical delivery systems struggle to overcome the complex microbial biofilm communities, and, as a result, there is minimal penetration of the antimicrobial, particularly in the deeper layers (reviewed in [7]). 

The benefits of using microneedles devices for transdermal drug delivery are greatly acknowledged. With a varying number of micron-sized needles, the microneedle patches can be loaded with drugs in the coating, inside, or even made to pass through hollow needles. Transdermal delivery of drugs using microneedles allows their passage across the stratum corneum layer, resulting in an incremented action onset and better patient compliance. Besides, they are easy to use, even by the patient itself, and have an improved drug permeability compared to other transdermal drug delivery devices (e.g., transdermal patches, topical creams, emulsions and ointments) [8]. Different materials or combinations can be used to produce microneedles (e.g., metal, biopolymers, metal-organic frameworks, polymer-ceramic) for transdermal drug delivery [9,10,11,12]. Besides the type of material used for fabrication, the microneedles need to perforate the skin, and this is highly affected by the geometry of microneedles, including height, width, shape, and mechanical characteristics. Current microneedle studies have mainly focused on the delivery of insulin [8,13,14] and vaccines [15,16], antibodies for cancer immunotherapy [17], and high molecular weight drugs [18]. A few microneedle studies have been published that aim to control bacterial and fungal biofilms, and have mainly focused on delivering antibiotics [19], antifungal agents [20], carvacrol [21], silver nanoparticles [22], elevating oxygenation [23]. 

Phages are bacterial viruses, and their discovery dates back to the early 1900s, and have many advantages over antibiotics, which include: targeted specificity without affecting the commensal flora, no undesired side effects, self-replicating nature as long as the host bacterium is present, multiplication at the infection site, killing multidrug-resistant bacteria, and a single therapy can target different pathogens if phage cocktail formulations are used [7,24]. Their clinical use under the umbrella of the Declaration of Helsinki (World Medical Association, Ethical Principles for Medical Research Involving Human Subjects) has increased significantly in recent years [7], and many countries have expanded schemes for non-approved therapeutics when no comparable or alternative therapy option is available [25,26,27]. Although phages possess interesting characteristics and are highly effective toward exponentially growing bacterial cultures, not all phages destroy cells when the host resides in biofilms. A few phages have good antibiofilm characteristics because they possess depolymerase activity towards capsular polysaccharides (CPS), exopolysaccharides (EPS) or lipopolysaccharide (LPS), all of which hold central functions related to biofilm production, virulence, and in the phage-host interaction process [28]. Phages not possessing depolymerase activity struggle to diffuse through the extracellular polysaccharide matrix, which acts as a barrier against the diffusion of phages [29,30]. Thus delivering phages without depolymerases throughout the different biofilm layers using microneedle approaches can be advantageous. 

**Scheme 1:**
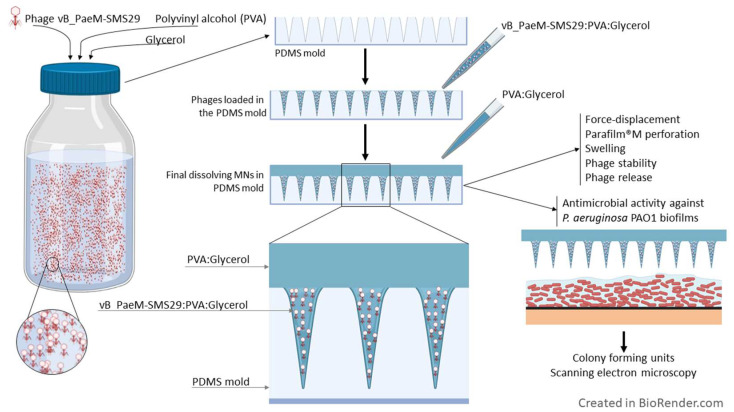
Overview of the experiments performed to characterize microneedles loaded with phages and evaluate their efficacy against biofilms.

This work focuses on dissolving microneedles fabricated using polyvinyl alcohol (PVA) to deliver the *Pbunavirus* vB_PaeM-SMS29 to *P. aeruginosa* PAO1 biofilms (Scheme 1). Two microneedle arrays producing geometrically different needles were tested to assess if the geometry might influence the distribution of phages throughout the biofilm layers and result in altered antimicrobial effects. We aim to establish proof that the delivery of phages using dissolving microneedles can be a good strategy for biofilm-related infection sites allowing phages to overcome the challenges posed by the challenging EPS matrix. 

## 2. Materials and Methods

### 2.1. Bacteria and Phage

*P. aeruginosa* PAO1 (DSM 22644) was used in this study. The strain was grown at 37 °C in broth or solid TSB medium (TSB + 1.2% *w*/*v* of agar). The *Pseudomonas* phage vB_PaeM-SMS29 (SMS29) used in this study has been previously characterized [31]. 

### 2.2. Phage Propagation and Titration

Phage SMS29 was amplified using the plate lysis and elution procedure [32]. The phage was titrated according to a previously described protocol using TSA plates and the host in TSB top-agar [TSB + 0.6% *w*/*v* agar] [33]. The tail-associated proteins were analyzed with HHPRED [34] to identify potential depolymerase domains.

### 2.3. Materials

Different polyvinyl alcohols (PVA, Sigma-Aldrich, St. Louis, MO, USA) varying in molecular weight and hydrolysis degree were tested (Table 1). The different PVA [final concentration 10% *w*/*w*] were hydrated in SM buffer [5.8 g·L^−1^ NaCl, 2 g·L^−1^ MgSO_4_·7H_2_O, 50 mL·L^−1^ of 50 mM Tris/HCl (pH 7.5) with 5 mL·L^−1^ of 2% *w*/*v* gelatin], under agitation (350 rpm) at room temperature (RT) for 4 h. Afterwards, the PVAs were dissolved under agitation (350 rpm) at 85 °C or 90 °C, according to their hydrolysis degree, until complete dissolution (approximately 2–3 h). The temperature of the PVA solutions was allowed to reduce to 64 °C before the addition of phages in order not to inactivate them. PVA containing phage SMS29 were produced with 10% *v*/*v* of phages.

### 2.4. Phage Stability in Liquid Polyvinyl Alcohol (PVA)

Phage SMS29 stability studies were performed in liquid PVA, PVA with polyvinylpyrrolidone (PVP) [1:7 *v*/*v* PVP:PVA], and PVA with glycerol 0.1% *v*/*v*. Briefly, 10% *v*/*v* of phage (final concentration of approximately 1 × 10^8^ plaque forming units (PFU)·mL^−1^) was added to the solutions (10% *w*/*w* final polymer concentration) and stored at 4 °C. Samples were removed for five days for phage titration as previously described [33]. 

### 2.5. Microneedle Array Fabrication

In-house microneedle arrays were produced as described previously [35]. In brief, silicon microneedles were microfabricated by sequential isotropic-anisotropic deep reactive ion-etching. Firstly, a silicon dioxide mask was patterned in a 1000 µm thick silicon wafer by lithography. The silicon substrate was then isotropically etched using SF6 (STPS Pegasus, to outline the needle tip shape. A second lithography was performed to protect the needle tip. A thick metal alloy (AlSiC9) was sputtered onto samples (Timaris FTM), and, after, a wet resist strip, the final height of the microneedles was achieved by anisotropic DRIE, using the standard Bosch process. The silicon wafer was diced into 11 × 11 arrays. The microneedle characterization was performed by scanning electron microscopy (SEM, Quanta FEG 650, FEI Europe B.V., Eindhoven, The Netherlands) and profilometry. 

### 2.6. Polydimethylsiloxane Molds

Two types of polydimethylsiloxane (PDMS) molds were used in this work. INL PDMS molds were those obtained by replica molding of the in-house microfabricated silicon masters as described previously [35,36]. The silicon masters were submitted to vapor silanization with trichloro(1H,1H,2H,2H-perfluorooctyl)silane as described before [36]. According to the manufacturer’s instructions, PDMS (Sylgard^®^184, Dow Corning, Midland, MI, USA) was mixed at a 10:1 *w*/*w* elastomer to curing agent ratio. PDMS molds were obtained after pouring PDMS in the silicone master, degassing under vacuum for 10 min, and curing at 65 °C for 1 h 30 min. The other PDMS molds were acquired from BlueAcre Technology (Dundalk, Ireland). These BlueAcre PDMS molds consist of 11 × 11 needles with a height of 600 µm, a base of 300 µm, and 600 µm from tip to tip. 

### 2.7. Polyvinyl Alcohol Microneedle Fabrication

Polyvinyl alcohol (PVA, Mowiol 4:88) was used to fabricate MNs, with and without phage SMS29, using a molding technique. Phage in SM buffer was mixed with PVA 4:88 with 0.1% *v*/*v* of glycerol (PVA_G_) to have a final concentration of 1 × 10^9^ PFU/mL. Twenty five µL of this mixture were placed in the PDMS molds, and then vacuum was applied (Agilent Technologies IDP3, Santa Clara, CA, USA). The molds were centrifuged at 1000 rpm (RT, 10 min, Universal 320, Hettich GmbH & Co. KG, Tuttlingen, Germany) to fill all needle cavities and eliminate entrapped air bubbles. Additional 50 µL of the mixture were placed on the PDMS molds, and the vacuum and centrifugation steps were repeated. The volume on the mold surface surrounding the MN area was removed with adhesive tape (3M), and the MNs were dried for 2 h at 40 °C in a ventilated oven (Termaks B8054, Bergen, Norway). After, PVA_G_ without phages was placed on the molds (100 µL) and dried for 2 h at 40 °C in a ventilated oven to create a thicker upper area for easier handling of the MNs. MNs in their respective PDMS molds were stored in vacuum-sealed bags at 4 °C or RT until their manual removal from the PDMS molds for further use. According to their fabrication place, the MNs were designated as INL (International Iberian Nanotechnology Laboratory) and BA (BlueAcre).

### 2.8. Phage Stability and Release from PVA_G_ MNs

The stability of phage SMS29 in the MNs was assessed over time after storage at 4 °C and RT. Briefly, MNs containing phages were removed from the PDMS molds and placed in 10 mL of SM buffer for 24 h at 37 °C under agitation (120 rpm) (Advanced 3500 Orbital Shaker, VWR). After 24 h, the solutions were vortexed thoroughly and serially 10-fold diluted. The release of phages was studied using a procedure described previously [37] with some modifications. Briefly, the 11 × 11 MNs were placed in 10 mL of SM buffer and incubated at 37 °C with agitation (120 rpm, Advanced 3500 Orbital Shaker, VWR). Samples (100 µL) were taken every minute until 5 min, every 5 min between 5 min and 20 min, and every 10 min until 60 min, and were serially diluted and plated. The volume was replenished with 100 µL of SM buffer at each time point. Petri dishes with serial dilutions were incubated for 16 h at 37 °C. The number of phages retrieved after the stability assay and during the release experiments was determined as described previously [33]. The data on phage release studies present the cumulative concentration of phages released over time. Two independent experiments were performed in triplicate.

### 2.9. Determination of the Swelling and Solubility Characteristics

Swelling and solubility studies were performed with films instead of MNs. PVA solutions dissolved as described above were poured (20 mL) onto 90 mm Petri dishes and degassed. The films were dried at 40 °C (Termaks B8054, Bergen, Norway), cut into 2 × 2 cm pieces, placed in a desiccator for 24 h, and after, the weight was measured (W_0_). The films were then placed in distilled water, and samples were taken every 2 min. The excess water was removed carefully using tissue paper, and the weight of the sample was measured (W_1_). The wet films were dried at 40 °C and placed in a desiccator, and the weight was measured (W_2_). The degree of swelling (%) and the solubility (%) were determined as follows: Swelling (%) = (W_1_ − W_0_)/W_1_ × 100, Solubility (%) = (W_0_ − W_2_)/W_2_ × 100. Two independent experiments were performed in duplicate. 

### 2.10. Optical and Scanning Electron Microscopy (SEM) of the MNs

The in-house INL master mold and PVA_G_ MNs (before and after force-displacement and perforation studies) were visualized by SEM (Quanta FEG 650, FEI Europe B.V., Eindhoven, The Netherlands) after gold sputtering (Leica Microsystems, EM ACE200, Wetzlar, Germany). Also, MNs were visualized with a wide-field upright optical microscope (Nikon—Eclipse Ni-E, Tokyo, Japan) coupled with a one-color camera (DS-Fi2, Nikon, Tokyo, Japan). 

### 2.11. Transmission Electron Microscopy (TEM) of the Phage

Phage particles were centrifuged (1 h, 25,000× *g*, 4 °C) and the pellet was washed twice with tap water. The samples was centrifuged once more (1 h, 25,000× *g*, 4 °C). Phages were deposited on 400 mesh copper grids and stained with 2% (*w*/*v*) uranyl acetate (pH 4.0). The grids were imaged at 200 kV using a JEM-2100-HT electron microscope (JEOL, Tokyo, Japan).

### 2.12. Force-Displacement 

The MNs were subjected to axial force mechanical studies using a force-displacement system (Tricor Systems Inc^®^, Elgin, IL, USA, Model 925). Briefly, a metallic probe was used, and the height aligned before any force was applied. MNs were glued to a plastic support with double-sided tape. The metallic probe moved down at 1.25 mm/s at a Fmax force of 3600× *g* until touching the array. The force-displacement plots were divided by the number of MNs. Five to six MNs of each geometry were analyzed.

### 2.13. Perforation Studies

Parafilm^®^M (PM-996, Bemis™ Parafilm™ M Laboratory Wrapping Film, Oshkosh, WI, USA) was folded in six layers and perforated with the different MNs. The thickness of the layers was measured with a Schut electronic outside micrometer (0.1–2.5 mm; 0.001 mm). The number of pores in the different layers was measured as previously described [38] using a wide-field upright optical microscope (Nikon—Eclipse Ni-E, Melville, NY, USA). For each type of MNs, 10–15 arrays were used. 

Porcine skin excised from the abdomen was generously supplied by ICVS-Life and Health Sciences Research Institute (Braga, Portugal). The hair was removed using scissors, and the fat was excised using a scalpel. Samples were then stored in vacuum-sealed bags at −20 °C and thawed only before use. The polymeric MNs were pressed onto the skin (thumb pressed) and removed after 5 min. The microneedle insertion sites were stained with Trypan blue 0.4% (*v*/*v*) (Sigma-Aldrich), the excess stain was removed with a wipe, and the skin samples were photographed. 

### 2.14. Antibiofilm Activity

The antibacterial activity of free phage SMS29 and phage-loaded MNS was assessed in 48 h-old biofilms of *P. aeruginosa* PAO1 produced using the colony biofilm procedure [39], with some modifications. Briefly, overnight cultures were adjusted to approximately 1 × 10^9^ colony forming units (CFU)·mL^−1^ and diluted 10-fold. Sterile polycarbonate membrane (Whatman^®^ Nuclepore^TM^ Track-etched, Maidstone, UK, 25 mm diameter, black, 0.2 μm pore size) were cut to the size of the MN arrays and placed on TSA plates with the shiny side facing up, and inoculated with 20 µL of this culture. The polycarbonate membranes were incubated at 37 °C, under static conditions, for 24 h and transferred to fresh TSA plates and incubated for another 24 h at 37 °C. Free phages (50 µL, 1 × 10^9^ PFU·mL^−1^) or SM buffer (50 µL) in the case of control samples were added to the membranes and incubated at 37 °C with samples removed at different time points for CFU determination. Unloaded and phage-loaded MNs were placed on the 48 h-old biofilms formed on the polycarbonate membranes. Also, a control with only *P. aeruginosa* PAO1 was analyzed. The TSA plates were incubated at 37 °C, and samples were removed for CFU determination after 8 h and 24 h. The polycarbonate membrane was removed at each sampling point and placed in 10 mL of sterile saline (0.9% *w*/*v* NaCl). Also, the TSA below the polycarbonate membrane was removed using a 5 mL syringe (Terumo^®^, Tokyo, Japan, Luer slip tip), which was previously cut to remove the tip and sterilized by autoclaving (121 °C, 15 min). The TSA block was removed and added to a new 10 mL of sterile saline to determine if this contained cells. The samples were vortexed, serially diluted in saline containing 10 mM ferrous ammonium sulfate [31], and plated in TSA for CFU determination. Total cell counts were determined as the sum of the cells in the polycarbonate membranes and the cells in the TSA plug. Three independent experiments were performed in duplicate. Surviving cells (20 colonies from each independent experiment) were collected after 24 h of treatment with free phages and SMS29-loaded onto MNs, and their susceptibility to the stock phages was evaluated as described previously [40]. 

### 2.15. Statistical Analysis

Statistical analysis of the results was performed using GraphPad Prism 6 (GraphPad Software, La Jolla, CA, USA). The independent experiments and the results are presented as mean ± standard deviation (SD). Differences between control and treated biofilms were assessed using one-way ANOVA followed by Tukey’s multiple comparison statistical tests. Differences were considered statistically significant if *p* < 0.05 (95% confidence interval).

## 3. Results and Discussion

### 3.1. Phage vB_PaeM-SMS29

**Figure 1:**
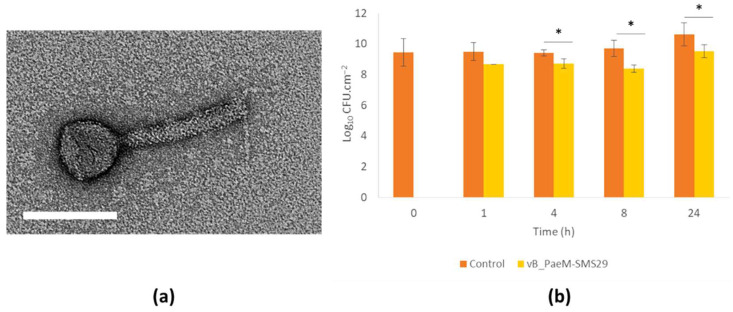
Phage vB_PaeP-SMS29 and its efficacy against 48 h-old *P. aeruginosa* PAO1 biofilms when delivered in a free form. (**a**) electron microscopy of vB_PaeM-SMS29, scale bar = 100 nm; (**b**) Number of cells (log_10_ CFU·cm^−2^) after different treatment periods with phage vB_PaeM-SMS29. * statistically different (*p* < 0.05).

Phage SMS29 was previously isolated from raw sewage and fully characterized [31]. It belongs to the *Pbunavirus* genus of the *Caudoviricetes* class, having a capsid of 70.0 ± 4.2 nm and a tail with a length of 139.0 ± 1.5 nm (Figure 1a). 

In this work, we used the colony-biofilm procedure to grow *P. aeruginosa* PAO1 on the surface of polycarbonate membranes for 48 h. This methodology forms static biofilms with a thicker EPS matrix, promoting bacterial growth by consumption of the nutrients present in the TSA plates (below the polycarbonate membrane). This method is, in our opinion, more appropriate to produce biofilms resembling those present in chronic wounds than biofilms produced in microtiter plates. The treatment with free phage SMS29 was performed for 24 h (Figure 1b). SMS29 was not very capable of reducing biofilm cells, resulting in a maximum reduction of 1.31 log_10_ CFU·cm^−^^2^ compared to the control after 8 h of treatment. However, these reductions are slightly higher than the antibiofilm efficacy of other Pbunaviruses. For instance, phage vB_PaeM_SCUT-S2 caused cell reductions in 24 h-old *P. aeruginosa* biofilms of approximately 50% and 69% after 4 and 24 h of phage challenge (less than 1 log reduction) [41], while phages vB_PaeM_USP_1, vB_PaeM_USP_2, vB_PaeM_USP_3, vB_PaeM_USP_18 and vB_PaeM_USP_25 reduced *P. aeruginosa* biofilms by 1 log_10_ CFU·cm^−^^2^, respectively [42]. Furthermore, by the end of the 24 h treatment, 15% of the surviving colonies had acquired resistance to SMS29. This percentage is slightly lower than the one reported for the surviving colonies following biofilm treatment with the *Pbunavirus* vB_PaeM_CEB_DP1, where 26% of the survivors had become resistant [43].

A search for depolymerase domains in the tail-related proteins of SMS29’s genome (ORFs SMS29_031 and SMS29_045) showed that none of these proteins had domains with depolymerase function. 

The poor antibiofilm efficacy allied to a lack of depolymerase activity makes this phage a good candidate for loading onto microneedles to assess if this delivery option allows its distribution throughout the 3D biofilms and enhances *P. aeruginosa* killing. 

### 3.2. Selection of the PVA for MN Fabrication

PVA dissolves at temperatures >80 °C, and, therefore, it is important to evaluate if the solution can be cooled to 65 °C and if it remains liquid so that phage SMS29 can be incorporated. Unfortunately, PVAs with high molecular weights and viscosities, when cooled to 65 °C, resulted in partial gelation (Table 1) and had to be discarded since their use would hamper the solution’s proper homogenization, resulting in unequal distribution of phages in the fabricated MNs. 

**Table 1:** Polyvinyl alcohols (Sigma-Aldrich) and their characteristics.

Polyvinyl Alcohol (PVA)	Characteristics (MW, Hydrolysis Degree, Viscosity)	After Cooling to 65 °C	Film after 24 h at 40 °C
Mowiol 4-98	MW 27,000, 98–99 mol% hydrolyzed, 4–5 mPa·s	Liquid	Slightly wrinkled film
Mowiol 4-88	MW 31,000, 86.7–88.7 mol% hydrolyzed; 3.5–4.5 mPa·s	Liquid	Uniform film
31,000–50,000	MW 31,000–50,000, 5.4–6.5 cP; 99+% hydrolyzed	Liquid	Slightly wrinkled film
Mowiol 8-88	MW 67,000, 86.7–88.7 mol% hydrolyzed; 7–9 mPa·s	Two phases	Not possible to use
85,000–124,000	MW 85,000–124,000, 28–32 cP; 99+% hydrolyzed	Two phases	Not possible to use
Mowiol 18-88	MW 130,000, 86.7–88.7 mol% hydrolyzed; 16–20 mPa·s	Two phases	Not possible to use
146,000–186,000	MW 146,000–186,000, 55–65 cP; 99+% hydrolyzed	Two phases	Not possible to use

Films of the PVA solutions that remained liquid at ≤65 °C were further prepared, and based on the uniform aspect, PVA 4-88 was selected for further studies (Figure S1). After its selection, it was important to verify if this PVA could somehow compromise the viability of phage SMS29. It was observed that the PVA 4-88 solution decreased the phage concentration already after 3 days when stored at 4 °C (Figure S2). Therefore, the addition of glycerol (0.1% *v*/*v*) and PVP (1:7 *v*/*v* ratio) was tested in order to improve the stability by increasing plasticity and reducing the crystallinity of the PVA molecules.

Glycerol is commonly used to improve phage preservation under different storage conditions [44] and has been shown to aid in phage diffusion, improving the size of pin-hole phage plaques, allowing a better determination of plaque forming units (PFU) [45]. The addition of 0.1% (*v*/*v*) of glycerol to PVA 4-88 maintained the phage concentration higher than PVA 4-88 alone, while the addition of PVP significantly decreased the concentration of phage SMS29, even when combined with 0.1% *v*/*v* of glycerol (Figure S2). A previous study using PVP to encapsulate an E. coli phage in nanofibers reported no loss in phage titers [46]. However, the use of PVP for encapsulating S. aureus phage ISP resulted in a partial inactivation before freezing and in a complete inactivation after lyophilization [47]. 

The swelling and dissolution of the different combinations of PVA, PVP or glycerol were evaluated (Table 2). 

The maximum swelling percentage before the disintegration of the samples started varied slightly between 61.9% (PVA:glycerol) and 67.1% (PVA:PVP). Although the addition of 0.1% (*v*/*v*) glycerol to PVA and PVA:PVA resulted in marginally lower degrees of swelling compared to PVA and PVA:PVA without glycerol, these were not statistically significant. Other studies have added glycerol to PVA hydrogels and PVA:PVP patches and observed a reduction in swelling compared to samples without glycerol [48,49]. This decrease is explained by glycerol molecules occupying the space around PVA’s hydroxyl groups, forming crosslinks between the PVA molecules, limiting the interactions between PVA and the water molecules [48]. However, the percentages used in both studies were above 0.1% (*v*/*v*) (0.75–3% (*v*/*v*) glycerol for PVA hydrogels and 0.37–1.5% (*v*/*v*) glycerol PVA:PVP patches). The increase of glycerol concentration would render the MNs fabricated in our work impossible to use as they would become too bendable, not being able to perforate the skin. 

### 3.3. Microneedle Characterization 

**Figure 2:**
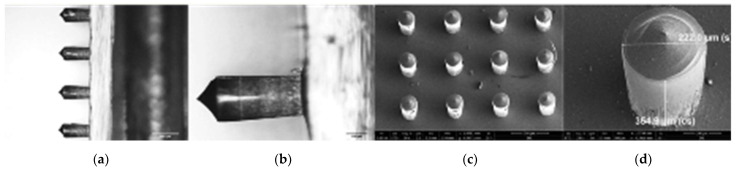
The in-house produced MNs visualized by optical microscopy (**a**,**b**, lateral view) and SEM (**c**,**d**, top view). Ruler in (**a**,**c**) is 500 µm, and in (**b**,**d**) is 100 µm.

The in-house fabricated silicon MNs were visualized by SEM (Figure 2). These cylinder-type MNs with a conical tip had a total height of 545.87 ± 48.76 µm and a diameter of 221.27 ± 1.48 µm. The cylinder part had a height of 356.83 ± 13.60 µm and a tip-to-tip width of 600 µm. 

**Figure 3:**
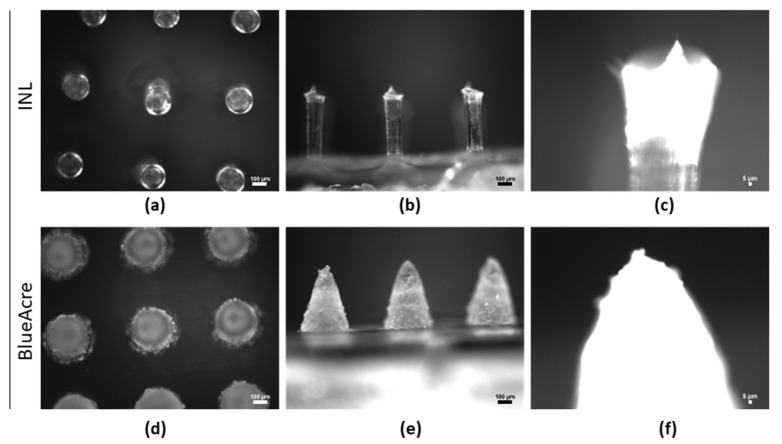
Top and lateral view of the INL and BlueAcre MNs fabricated using PVA dissolved in SM buffer with glycerol 0.1% (*v*/*v*) and visualized with a wide-field upright optical microscope. (**a**,**d**) top view imaged with ×5 objective; (**b**,**e**) lateral view imaged with ×5 objective, (**c**,**f**) lateral view of the needle tip with ×20 objective. The ruler in (**a**,**b**,**d**,**e**) is 100 µm, and in (**c**,**f**) is 5 µm.

The polymeric MNs fabricated with PVA 4-88 with 0.1% *v*/*v* of glycerol (from herein designated as PVA_G_) were also observed by optical and SEM microscopy (Figure 3).

The PVA_G_ MNs had average heights and base diameters of 518.16 ± 48.06 µm and 205.00 ± 13.54 µm for the INL MNs, and 514.86 ± 7.42 µm and 294.78 ± 14.27 µm for the BA MNs, respectively. The height and base diameters were reduced by approximately 5.08% and 7.32% in the INL MNs compared to the silicon MNs (Figure 2) and 14.19% and 1.74% for BA MNs compared to the purchased molds’ specifications. Differences in height and base diameters between master molds and polymer MNs are common due to the dehydration of the polymer and have been reported for different dissolvable, swellable and biodegradable polymers [50,51]. For instance, Demir et al. used pyramidal-shaped MN (900 µm height and 250 µm base diameter) and six polymers to produce MNs. In their study, they reported the lowest height of 826.89 ± 15.09 µm using 3% (*w*/*w*) chitosan MNs (8.12% reduction) and the most approximate to the master mold was a height of 899.89 ± 1.66 µm with poly (D, L-lactic-co-glycolic acid) (PLGA) (0.01% reduction) [50]. Also, the base width varied, being the most distant to the master mold using 10% (*w*/*w*) of sodium alginate (206.70 ± 2.94 µm, ~17.32 reduction) to the most similar when fabricated with PLGA (250.39 ± 1.12 µm). Also, Bonfante et al. described reductions in height and base diameter of approximately 10–19% with hyaluronic acid and carboxymethyl cellulose [51].

**Figure 4:**
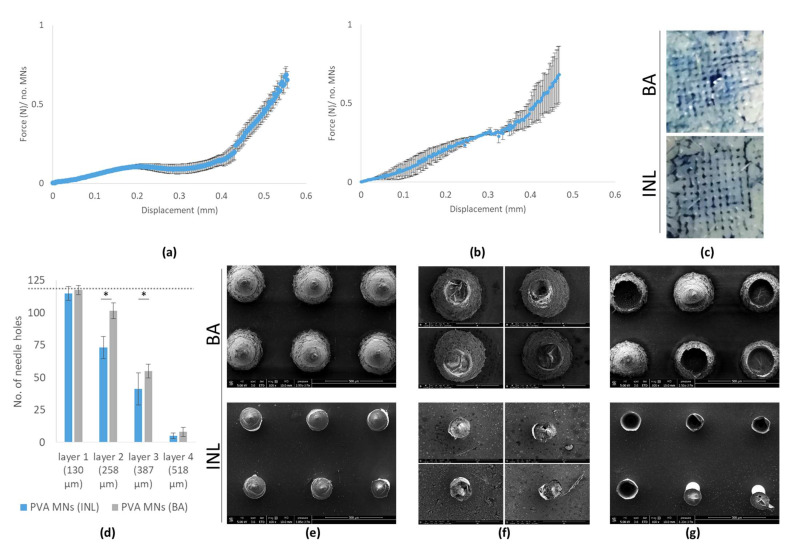
Force-displacement and Parafilm^®^M perforation using the PVA MNs fabricated with the different molds. (**a**) force-displacement of the PVA_G_ INL MNs; (**b**) force-displacement of the PVA_G_ BlueAcre (BA) MNs; (**c**) porcine skin perforation test stained with Trypan blue (0.4% *v*/*v*); (**d**) Parafilm^®^M perforation test, dashed line corresponds to the maximum of needles in the arrays (121 needles), and the average thickness of the layers was measured with a Schut electronic outside micrometer (0.1–2.5 mm; 0.001 mm), * statistically significant (*p* < 0.05); (**e**) SEM micrographs of PVA_G_ MNs (INL and BluAcre) after fabrication, (**f**) SEM micrographs of PVA_G_ MNs (INL and BluAcre) after fabrication force-displacement, (**g**) SEM micrographs of PVA MNs (INL and BluAcre) after Parafilm^®^M perforation tests. Ruler in (**e**,**g**) is 500 µm, and in (**f**) is 300 µm.

The mechanical characteristics of the INL and BA MNs were investigated by force-displacement and Parafilm^®^M perforation (Figure 4). The results show that MN geometry has a significant role in the tested properties. For example, the force for needle failure of INL MNs was 0.1 N, which increased to 0.3 N for those produced with the BA mold (Figure 4a,b). This points to a greater resistance of the conical shape (BA) against the compressive force than the cylindrical with a sharp tip (INL). Differences in the mechanical behavior of MNs have been reported, for instance, with PVP MNs having circular obelisk, pyramidal, and beveled-circular obelisk geometries [52]. According to the authors, the beveled-circular obelisk MNs were the most resistant, followed by the circular obelisk and the pyramidal. Also, Gittard et al. showed differences in three conical geometries varying in base diameter and height ratios [A = 250:750 (weakest), B = 300:750, C = 250:500 (strongest)] [53]. MNs imaged after force-displacement tests show that BA MNs were flattened while INL MNs broke (Figure 4f). 

MN perforation studies were carried out in porcine skin and Parafilm^®^M layers (Figure 4c,d). Freshly porcine skin’s perforation showed that the needles perforated the skin and that the holes were positively stained with trypan blue (0.4% *v*/*v*) (Figure 4c). The Parafilm^®^M test has been proposed as a model membrane alternative to skins and allows verification of the number of needles perforating each Parafilm^®^M layer [54]. A significantly higher number of BA MNs perforated the different layers (Figure 4d). The number of holes caused by the BA MNs in the second (258 µm thickness) and third (387 µm) layers were reduced by 38% and 66%. SEM imaging following perforation studies showed that part of the MNs was not retrieved after their removal from the Parafilm^®^M (Figure 4g). Larrañeta et al. tested two 11 × 11 MNs made with a blend of Gantrez^®^S-97 (20% *w*/*w*) and PEG 10,000 (7.5% *w*/*w*) [54]. One was identical to our BA MNs (600 µm height, 300 µm base diameter), and the other produced MNs that were 900 µm in height and 300 µm in base width. In their study, Parafilm^®^M puncture tests showed that MNs with a 600 µm height resulted in an insertion depth between approximately 290 µm to 480 µm. These results are similar to those obtained with the BA MNs. Cordeiro et al. tested seven different geometries [55], including two geometries similar to those used in this work. In their Parafilm^®^M insertion tests, the number of holes in the different layers reduced immediately when the cylindrical MN with a conical tip was used. On the other hand, the number of holes using the conical MN decreased only after an insertion depth of 381 µm. These results are in agreement with those obtained in this work, where we show a greater perforation capability with the BA MN geometry than with the INL MNs. 

### 3.4. Phage-Loaded MNs

#### 3.4.1. Phage vB_PaeM-SMS29 Stability in MNS during Storage

**Figure 5:**
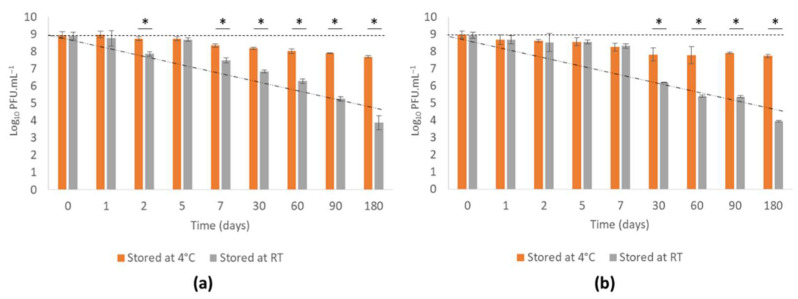
Stability of phage vB_PaeM-SMS29 in MNs after storage at 4 °C and room temperature (RT) for different periods up to 180 days. (**a**) BA MNs; (**b**) INL MNs. * statistically significant (*p* < 0.05). The dashed black line and the long dashed line with dots correspond to the concentration of vB_PaeM-SMS29 stored at 4 °C and RT in SM buffer.

Phage SMS29 stability in INL and BA MNs was assessed after storage for different times by determining the phage titers (Figure 5).

The concentration of SMS29 stored in SM buffer at 4 °C remained stable throughout the experiment (Figure 5, dashed line) but was significantly reduced when SMS29 in SM buffer was stored at RT (Figure 5, long dashed line with dots).

SMS29 loaded MNs stored at 4 °C hardly lost activity during the first days of storage, but after 180 days, the phage concentration had reduced by 0.95 log_10_ PFU·mL^−^^1^ (INL MNs) and 1.28 log_10_ PFU·mL^−^^1^ (BA MNs) compared to day 0. 

Storage at RT gradually decreased the concentration of SMS29, causing a maximum reduction of 4.87 log_10_ PFU·mL^−1^ (BA) and 4.76 log_10_ PFU·mL^−1^ (INL) by the end of the experiment (Figure 5). These decreases in concentration are related to the inactivation of some phages as observed by TEM (e.g., empty capsids and separation of the tails from the capsids, Figure S3). It is generally known that temperature plays a role in phage stability and activity. For instance, phage lysates and concentrated purified phage stocks stored in SM buffer hardly decrease in concentration when stored at 4 °C. For instance, phage PRD1 was stable for 22 years when stored at 4 °C in broth [56]. Furthermore, long-term storage of phages at room temperature is not usually endorsed. 

Overall, the phage-loaded MNs kept the phage concentration fairly high when stored at 4 °C for six months. These are important results since phage stability is extremely important in therapeutic applications. Furthermore, the brief stability of SMS29-loaded MNs stored at RT is important and can even facilitate shipping and short-term storage in non-refrigerated conditions. However, when phage stability is not fully investigated, it can compromise the outcome of the studies. Unfortunately, this has already occurred in a clinical trial—PhagoBurn. This trial had difficulties in manufacturing challenges and also the participant recruitment period was shortened, leading to a lower number of inclusions. The recruited participants were supposed to receive 12 *P. aeruginosa* phages at a concentration of 6 log_10_ PFU·mL^−^^1^, but the products applied were found to have only 1–2 log_10_ PFU·mL^−^^1^ due to a lack of stability [57]. This eventually contributed to the failure of the trial, and no conclusions regarding safety could be withdrawn from this clinical trial. 

#### 3.4.2. Phage vB_PaeM-SMS29 Release from MNs after Storage at Different Temperatures for Six Months 

**Figure 6:**
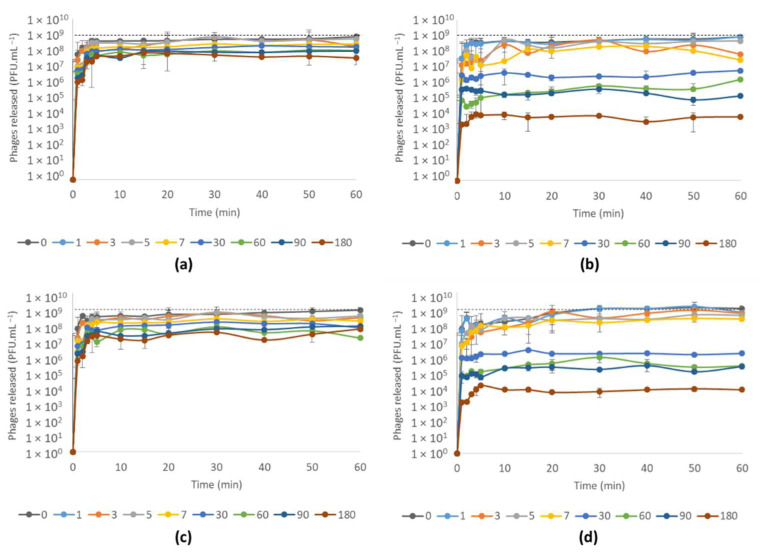
Release of phage vB_PaeM-SMS2s9 from PVA MNs after storage at 4 °C and room temperature (RT) for different periods up to 180 days. (**a**) SMS29 release from BA MN stored at 4 °C; (**b**) SMS29 release from BA MNs stored at RT; (**c**) SMS29 release from INL MNs stored at 4 °C; (**d**) SMS29 release from INL MNs stored at RT. The dashed lines correspond to the concentration of phages added to the fabricated MNs.

Phage release from the INL and BA MNs was performed after different storage periods for up to six months (Figure 6). 

The geometry of the MNs had no significant influence on the phage release profiles. The release of SMS29 from MNs stored at 4 °C was quite fast and reached high phage concentrations with barely any differences in the release profiles within the first days, with curves often appearing superimposed (Figure 6a,c). After longer storage periods, the concentrations of SMS29 decreased and the curves plateaued in inferior PFU values than added to produce the MNs (dashed lines). The release of SMS29 from MNs stored at RT showed a fast decline in phage concentration visibly more pronounced following 7 days (Figure 6b,d), corroborating the stability results (Figure 4). 

Previous works have encapsulated *P. aeruginosa* phages into different carriers, including hydrogels, liposomes, fibrin glue, polycaprolactone nanofibers for biomedical use [58,59,60,61]. For instance, *P. aeruginosa* phages PEV1 and PEV31 were encapsulated hydrogels formulated with PVA, and 77% of PEV1 and 76% of PEV31 were released after 1 h [62]. These results are close to the ones presented herein for samples stored at 4 °C and at RT during the first days (1–5 days). 

#### 3.4.3. Antimicrobial Efficacy of Phage vB_PaeM-SMS29-Loaded MNs

**Figure 7:**
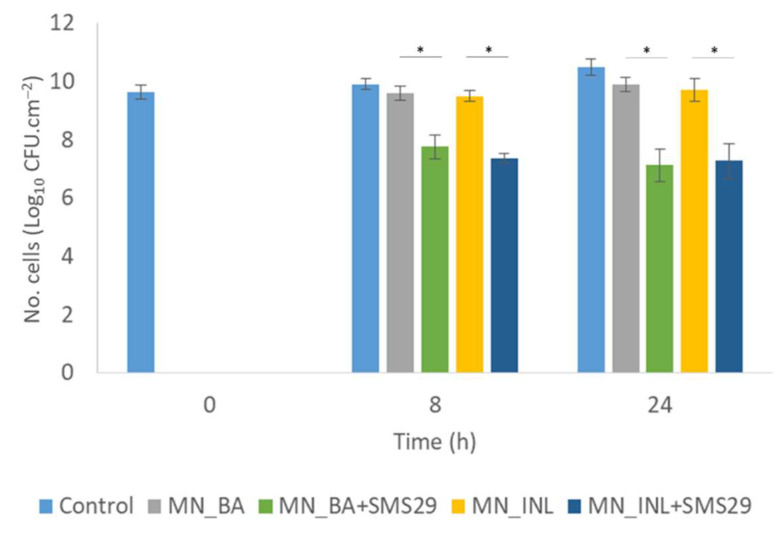
Evaluation of the antimicrobial efficacy of vB_PaeM-SMS29, delivered in buffer and loaded onto the INL and BA MNs, against 48 h-old *P. aeruginosa* PAO1 biofilms. The effect was evaluated after 8 and 24 h of treatment. * statistically significant (*p* < 0.05).

**Figure 8:**
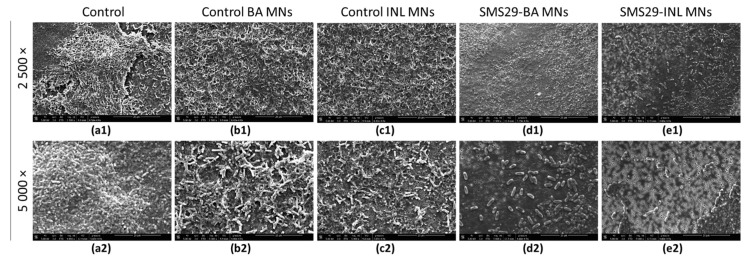
SEM micrographs of untreated and phage vB_PaeM-SMS29 treated (24 h treated) *P. aeruginosa* PAO1 biofilms. Biofilms after treatment for 24 h with (**a**) no phages and no MNs (control); (**b**) non-phage loaded control BA MN; (**c**) non-phage loaded control INL MN; (**d**) phage-loaded BA MN; (**e**) phage loaded INL MN; (**a1**–**e1**) biofilms imaged with a magnification of 2500×; (**a2**–**e2**) biofilms imaged with a magnification of 5000×.

The antimicrobial efficacy of phage SMS29 loaded onto the two MNs was evaluated against 48 h-old biofilms and compared to the control and unloaded MNs. The antimicrobial efficacy was evaluated by CFU counts and SEM (Figure 7 and Figure 8). 

The control and the non-phage-loaded MNs showed similar cell counts after 8 h and 24 h (Figure 7) (*p* > 0.05). On the other hand, the treatment of biofilms with phage SMS29 in INL and BA MNs decreased the viable biofilm cell counts significantly (*p* < 0.05). Although the results obtained do not vary according to the MN geometry used, we expected some differences. We hypothesized that a larger MN base diameter (BA MNs) would allow phages to reach and kill a higher number of cells due to the 90 µm larger base diameter in the BA MNs. After 8 h, the phages in MNs had reduced the number of cells by approximately 1.84 log_10_ CFU·cm^−2^ (BA) and 2.14 log_10_ CFU·cm^−2^ (INL) compared to their respective controls (*p* < 0.05). The number of cells continued to decrease, and after 24 h of treatment, they were 2.76 log_10_ CFU·mL^−2^ (BA) and 2.44 log_10_ CFU·mL^−2^ (INL) lower than in the controls. Overall, these reductions were significantly higher than those obtained when free SMS29 was used [1.31 log_10_ CFU·cm^−2^ (8 h) and 1.09 log_10_ CFU·cm^−2^ (24 h), Figure 1]. Furthermore, it is acknowledged that the effect is merely due to an enhancement of phage diffusion throughout the biofilm layers and not due to a mechanical dispersal of cells since this is not possible considering the colony biofilm model used. 

In terms of the susceptibility of the surviving cells to the SMS29 stock solution, the percentage of cells showing resistance after 24 h of treatment increased very slightly from 15% (free SMS29) to 16.7%. 

The 48 h-old biofilms treated with phages for 24 h treatment, and the respective controls were imaged by SEM (Figure 8). The control samples (Figure 8a1,2–c1,2) show areas fully covered with *P. aeruginosa*. On the other hand, the samples after treatment with phage-loaded MNs showed significantly fewer cells (Figure 8d1,2–e1,2), cell debris (visible in Figure 8d1,e1), and also footprints where bacteria used to be adhered (lighter areas, particularly in Figure 8e2), corroborating the CFU results (Figure 7).

## 4. Conclusions

In this work, we have encapsulated SMS29, a phage belonging to the *Pbunavirus* genus, in dissolving, biodegradable MNs. These MNs were made with PVA, a polymer frequently used in pharmaceutical applications due to its biocompatible and toxicologically safe characteristics. Storage at refrigerated conditions kept SMS29 in MNs at very high concentrations, and these started releasing the phage within the first minutes due to PVA’s dissolution. We have successfully validated the use of phage-loaded MNs for antibiofilm purposes, showing that this strategy enhanced the overall antimicrobial activity of this particular *Pbunavirus* compared to its free application to biofilms. This phage delivery strategy might, in the future, allow the use of other phages with poor antibiofilm activity for the treatment of topical biofilm-related wounds, particularly those colonized with antibiotic resistant pathogens. To our knowledge, this is the first study that incorporates a phage into dissolving MNs and provides a thorough characterization. Further studies using ex vivo and in vivo models and phage combinations may provide valuable knowledge for future phage therapy applications for biofilm-related skin infections.

## Figures and Tables

**Table 2:** Degree of swelling and solubility of PVA 4-88 combined with glycerol and PVP.

Sample	Degree of Swelling (%)	Time until Full Disintegration (min)	Dissolution (%) *
PVA	62.7 ± 18.7	7 ± 2	90.9 ± 5.3
PVA: 0.1% glycerol	61.9 ± 17.2	11 ± 2	95.9 ± 1.2
PVA:PVP	67.1 ± 35.7	9 ± 2	80.8 ± 10.4
PVA:PVP: 0.1% glycerol	62.0 ± 34.1	11 ± 2	90.9 ± 7.6

* measured at the end of the 15 min experiment.

## Supplementary Materials

The following supporting information can be downloaded at: https://www.mdpi.com/article/10.3390/v14050964/s1, Figure S1: The physical appearance of the films formed after casting and drying. (a) 4-98, (b) 4-88, (c) 31,000–50,000; Figure S2: Influence of polyvinyl alcohol (PVA), glycerol and polyvinyl pyrrolidone (PVP) on phage vB_PaeM-SMS29 viability; Figure S3: TEM micrographs of virion particles with empty capsids and capsids without tails attached.
