# Supplementary material for: Antibiofilm Efficacy of the Pseudomonas aeruginosa Pbunavirus vB_PaeM-SMS29 Loaded onto Dissolving Polyvinyl Alcohol Microneedles
Source: Viruses. 2022 May 5;14(5):964. doi: 10.3390/v14050964 (PMC9143888; doi:10.3390/v14050964)
Supplement: Supplementary file 1 [file viruses-14-00964-s001.zip › viruses-1697135-supplementary.pdf]

Supplementary material

Antibiofilm Efficacy of the *Pseudomonas aeruginosa Pbunavirus* vB_PaeM-SMS29 Loaded onto Dissolving Polyvinyl Alcohol Microneedles

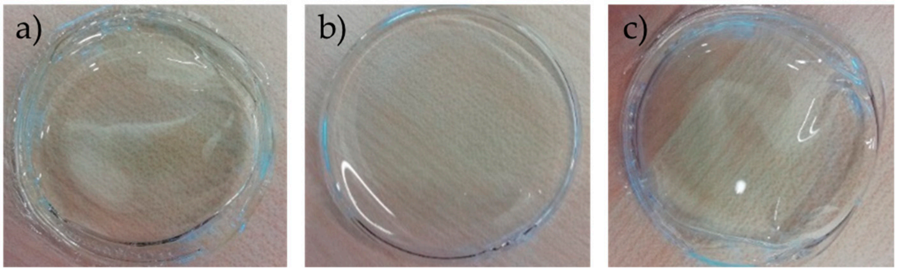

Figure S1. The physical appearance of the films formed after casting and drying. (**a**) 4-98, (**b**) 4-88, (**c**) 31,000–50,000.

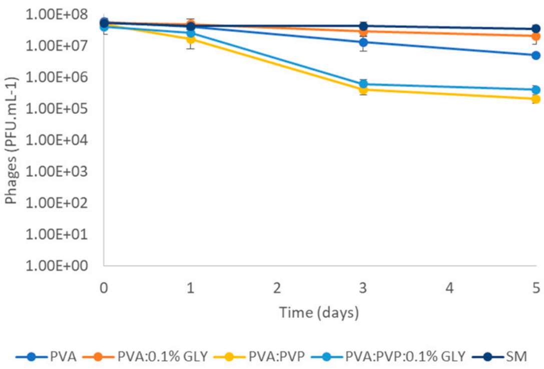

Figure S2. Influence of polyvinyl alcohol (PVA), glycerol and polyvinyl pyrrolidone (PVP) on phage vB_PaeM-SMS29 viability.

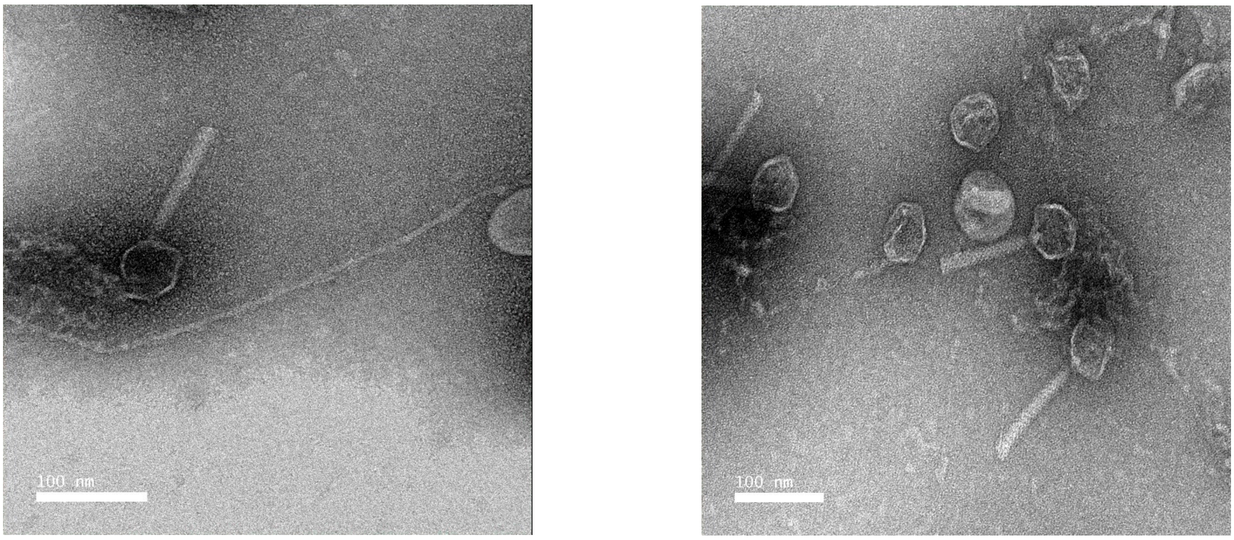

(**a**) (**b**)

Figure S3. TEM micrographs of virion particles with empty capsids (**a**) and capsids without tails attached (**b**). Ruler is 100 nm.
